# Gestational diabetes mellitus and the risk of autism spectrum disorder in offspring: a population-based retrospective cohort study

**DOI:** 10.3389/fcdhc.2026.1754571

**Published:** 2026-02-23

**Authors:** David Rubinshtein, Omri Zamstein, Tamar Wainstock, Eyal Sheiner

**Affiliations:** 1 Department of Obstetrics and Gynecology, Soroka University Medical Center, Ben-Gurion University of the Negev, Beer-Sheva, Israel; 2 Department of Public Health, Faculty of Health Sciences, Ben-Gurion University of the Negev, Beer-Sheva, Israel

**Keywords:** autism spectrum disorder, gestational diabetes mellitus, neurodevelopmental outcomes, population-based cohort study, pregnancy, retrospective cohort study

## Abstract

**Background:**

While several environmental and perinatal factors have been associated with the development of autism spectrum disorder (ASD), there is still much to uncover. In this study, we investigated the possible association between gestational diabetes mellitus (GDM), a condition that is becoming more widespread worldwide, and the risk of ASD.

**Methods:**

A population-based retrospective cohort study was conducted using data from a tertiary referral hospital and affiliated community clinics. ASD diagnoses were identified through centralized outpatient and hospital records and were established by qualified specialists in accordance with DSM-5 criteria during long-term childhood follow-up. The incidence of ASD in offspring was compared between pregnancies complicated by GDM, categorized as A1 (diet-controlled) or A2 (requiring pharmacologic treatment), and pregnancies without GDM. Cumulative incidence of ASD was estimated using Kaplan-Meier survival analysis, and a Cox proportional hazards model was applied to adjust for potential confounders.

**Results:**

Among 115,063 deliveries included in the study, 3,461 (3.0%) were complicated by GDM A1 and 1,164 (1.0%) by GDM A2. Overall, 767 offspring were diagnosed with ASD during childhood. Univariate analysis demonstrated a statistically significant association between GDM severity and ASD incidence (1.5% for GDM A2, 1.0% for GDM A1, and 0.6% for no GDM; p<0.001). Kaplan-Meier analysis demonstrated a significant difference in cumulative ASD incidence across GDM subtypes (log-rank p<0.001). However, after adjustment for confounders in a multivariable Cox model, neither GDM A1 nor GDM A2 was statistically significantly associated with ASD risk (aHR 1.18, 95% CI 0.83–1.66; aHR 1.56, 95% CI 0.95–2.56, respectively).

**Conclusion:**

Our findings suggest no statistically significant association between GDM and ASD in offspring after adjustment.

## 1 Introduction

Autism spectrum disorder (ASD) represents a major and growing public health concern, with recent U.S. data indicating a prevalence of approximately 1 in 30 children (1). ASD is widely recognized as a multifactorial neurodevelopmental disorder arising from complex interactions between genetic susceptibility and environmental influences (2–4). Given that ASD typically manifests early in life, alterations in brain development during the prenatal period are suspected to play an etiological role (3, 5, 6). Metabolic derangements during pregnancy, particularly diabetes mellitus, have gained attention for their potential impact on fetal neurodevelopment (7–9). Maternal hyperglycemia may impair normal brain maturation through pathways involving oxidative stress, inflammatory responses, mitochondrial dysfunction, and epigenetic modification (10–12).

Previous studies examining maternal diabetes and neurodevelopmental outcomes have suggested an increased risk of developmental delay and various neurobehavioral disorders, including ASD, among exposed offspring (13, 14),. A comprehensive meta-analysis by Ye et al. found that children born to individuals with diabetes during pregnancy had a 25–30% higher likelihood of developing ASD (14). However, the magnitude of this association varied substantially by diabetes subtype. The strongest and most consistent relationships have been reported for pregestational diabetes as well as for maternal metabolic dysregulation, including pre-pregnancy obesity, whereas the association with gestational diabetes mellitus (GDM) was weaker and often diminished after adjustment for multiple confounders. Interpretation of these findings is further complicated by considerable heterogeneity across studies, stemming from differences in study design, diagnostic definitions, and the timing of GDM diagnosis. Additional evidence suggests that both the duration and severity of maternal hyperglycemia may be key determinants of neurodevelopmental risk (15, 16).

Given the increasing global prevalence of both GDM and ASD (17–19) and the ongoing uncertainty regarding their potential link, we conducted a large population-based cohort study to further clarify the relationship between these two conditions.

## 2 Materials and methods

This retrospective cohort study included deliveries occurring between 2005 and 2017, with follow-up for ASD diagnoses extending through 2021. The study population included deliveries of patients insured by Clalit Health Services (CHS), the largest health maintenance organization in Israel. Because CHS provides universally accessible healthcare and its insured population reflects the general demographics of southern Israel (20), this cohort provides a representative sample of the region.

Pregnancies were classified into three groups according to treatment modality: diet-controlled GDM (GDM A1), medication-treated GDM (GDM A2), and pregnancies without GDM, which served as the reference group. Obstetric and perinatal data were obtained from the Soroka University Medical Center (SUMC) Perinatal Database, in which information is recorded shortly after delivery by attending obstetricians and verified by trained medical secretaries. Although diagnostic criteria for GDM evolved during the study period, classification based on treatment reflects clinically significant hyperglycemia and is less dependent on specific glucose thresholds and thus was chosen as the classification criterion. ASD diagnoses were extracted from CHS outpatient and hospital records and were established by developmental pediatricians, pediatric neurologists, child psychiatrists, or clinical psychologists, in accordance with DSM-5 criteria (21), requiring evidence of persistent deficits in social communication and interaction alongside restricted and repetitive behaviors, interests, or activities. The CHS database is routinely updated and has been validated in previous epidemiological studies for its reliability and accuracy in capturing clinical diagnosis (22). The study was conducted and reported in accordance with the Strengthening the Reporting of Observational Studies in Epidemiology (STROBE) guidelines.

**Figure 1:**
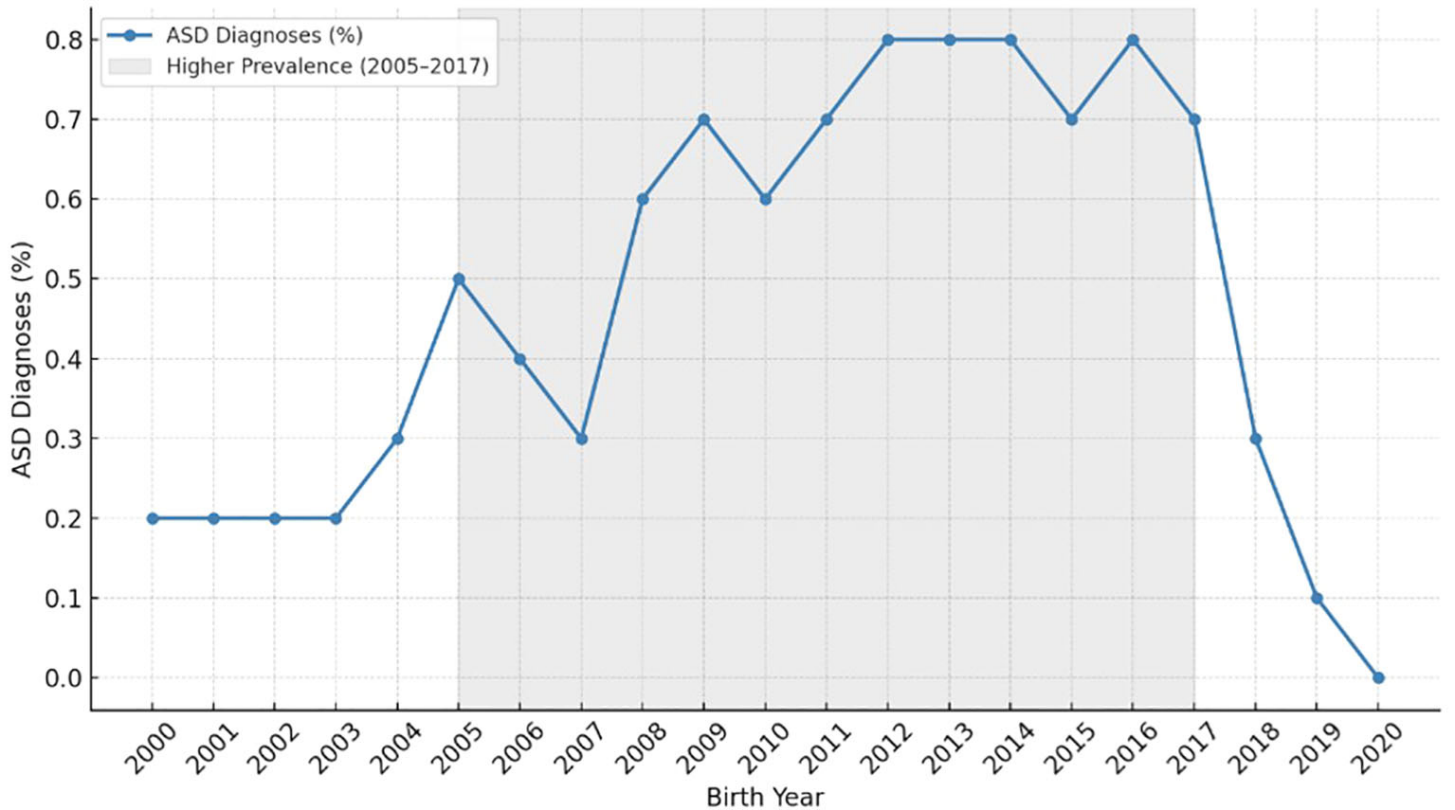
Distribution of autism spectrum disorder (ASD) diagnoses by birth year, with peak prevalence among births between 2005 and 2017.

Deliveries prior to 2005 were excluded due to limited diagnostic awareness of ASD, while those after 2017 were omitted to ensure sufficient follow-up time for reliable diagnosis by 2021 (23) (**Figure 1**). Pregnancies complicated by pregestational diabetes or diabetes first recognized early in pregnancy were identified based on documented diagnoses in the medical record and excluded from the analysis. Pregnancies complicated by major congenital anomalies and multifetal gestations were excluded as well. The participant selection process is illustrated in **Supplementary Figure S2**. Ethical approval was obtained from the Institutional Review Board of Soroka University Medical Center (IRB: 0357-19-SOR).

### 2.1 Statistical analysis

Statistical analysis was performed using SPSS for Windows (Version 29.0; IBM, Chicago, IL). Categorical variables were presented as frequencies and percentages, while normally distributed continuous variables were expressed as means with standard deviations. Comparisons across the three study groups (GDM A1, GDM A2, and non-GDM) were performed using the Chi-square test for categorical variables and one-way ANOVA for continuous variables. Cases involving perinatal mortality were excluded from long-term analyses. Kaplan–Meier survival curves were generated to evaluate the cumulative incidence of ASD diagnoses during childhood, and differences between the groups were assessed using the log-rank test. Covariate selection for the primary Cox model was guided by a directed acyclic graph (DAG) (**Supplementary Figure S1**). The primary model (**Table 1**) adjusted for baseline confounders available in the dataset (maternal age, ethnicity as a binary variable, pre-pregnancy obesity, smoking, fertility treatment, birth year, and child sex) to estimate the total effect of GDM on ASD risk. Pre-pregnancy obesity was defined as a binary variable based on documented clinical obesity status (BMI ≥30 kg/m²). Continuous pre-pregnancy BMI measurements were not consistently available in the dataset. A p-value of < 0.05 was considered statistically significant.

**Table 1:** Association between autism spectrum disorder in offspring and pregnancy complicated by GDM A1 or A2.

Outcome	No GDM (n=110,438)	GDM A1 (n=3,461)	GDM A2 (n=1,164)	p-value
ASD cases, n (%) (cases, %)	715 (0.6)	35 (1.0)	17 (1.5)	<0.001
Adjusted HR ^a^	1 (reference)	1.18 (95% CI 0.83–1.66, p = 0.34)	1.56 (95% CI 0.95-2.56, p = 0.076)	

^a^Adjusted for factors such as pre-pregnancy obesity, ethnicity, gender, fertility treatment, birth year and smoking.

## 3 Results

Among 115,063 deliveries included in the cohort, 3,461 (3.0%) were complicated by GDM A1 and 1,164 (1.0%) by GDM A2. Each subgroup was compared with pregnancies not complicated by GDM. Patients with GDM were generally older and had higher rates of hypertensive disorders during pregnancy. They were also more likely to conceive following fertility treatments and to undergo induction of labor. Pregnancies complicated by GDM, particularly GDM A2, were associated with earlier delivery, as well as markedly higher rates of cesarean delivery (48.9% vs. 14.4%) and preterm birth (<37 weeks; 14.8% vs. 6.8%). Neonates in the GDM groups had higher mean birthweight compared with those in the non-GDM group (**Table 2**).

**Table 2:** Baseline characteristics of the study population according to GDM status.

Characteristic	No GDM (n=110,438)	GDM A1 (n=3,461)	GDM A2 (n=1,164)	p-value
Maternal age at delivery, y (mean ± SD)	28.0 ± 5.7	32.0 ± 5.7	33.3 ± 5.7	<0.001
Ethnicity				<0.001
Bedouin, %	61.6	46.8	55.0	
Jewish, %	38.4	53.2	45.0	
Primiparity, %	24.9	25.6	17.3	<0.001
Pregnancy following fertility treatment, %	2.1	6.6	6.7	<0.001
Hypertensive disorder in pregnancy, %	3.6	9.1	15.9	<0.001
Gestational age at delivery, week (mean ± SD)	39.0 ± 1.9	38.7 ± 1.7	37.5 ± 1.8	<0.001
Induction of labor, %	17.8	29.4	35.5	<0.001
Non-reassuring fetal heart rate pattern, %	6.3	7.2	6.1	0.094
Cesarean delivery, %	14.4	30.1	48.9	<0.001
Gender				0.015
Female, %	49.4	47.7	46.2	
Male, %	50.6	52.3	53.8	
Preterm delivery (<37 weeks), %	6.8	7.6	14.8	<0.001
Birth weight, g (mean ± SD)	3,177 ± 512	3,357 ± 533	3,364 ± 614	<0.001
Low birth weight (< 2,500 g), %	7.3	4.6	5.6	<0.001
5-min Apgar < 7, %	0.6	0.7	1.1	0.105
Cord blood pH < 7, %	0.3	0.0	1.2	0.307
Perinatal mortality, %	0.8	0.4	1.5	<0.001

**Figure 2:**
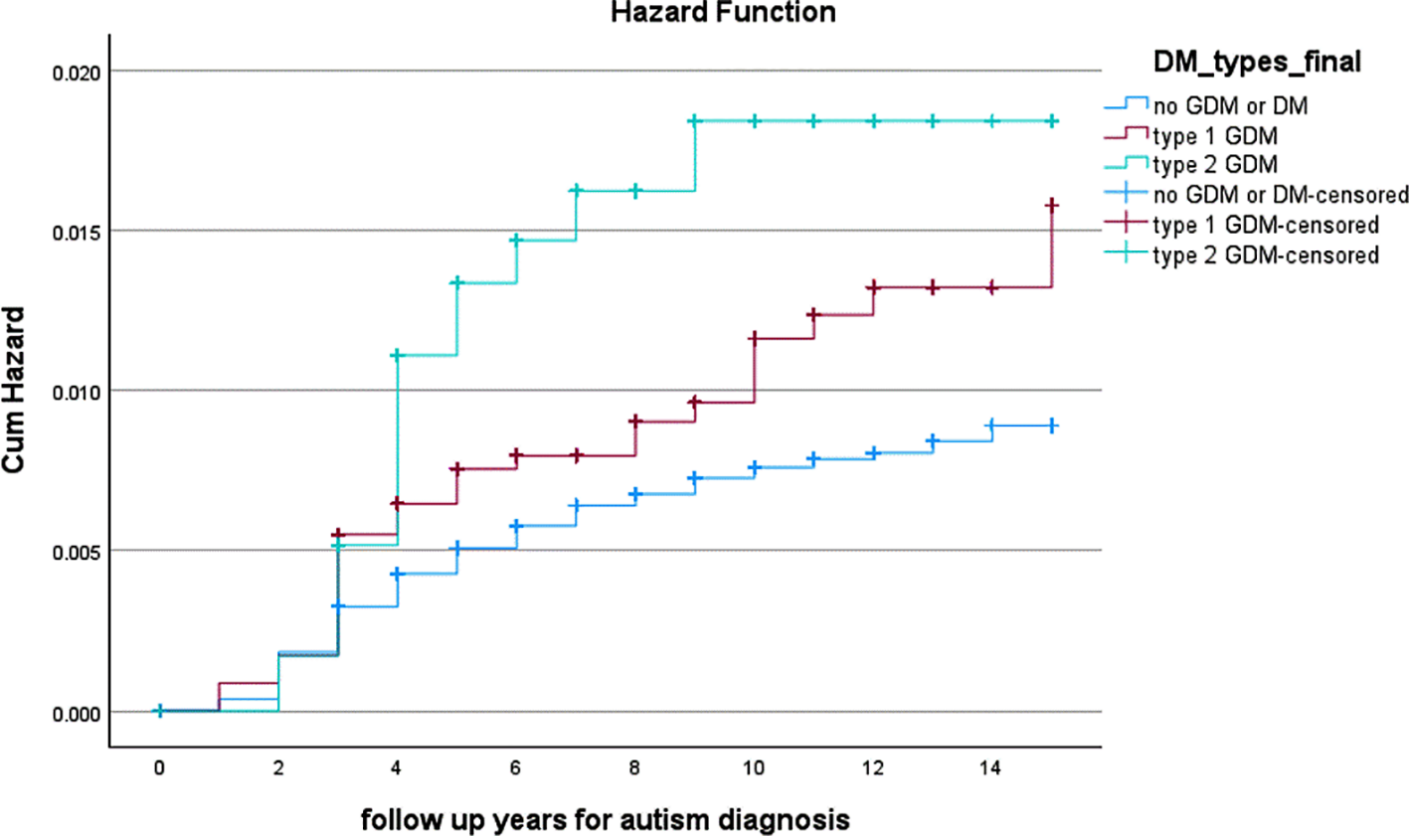
Cumulative incidence of autism spectrum disorder diagnoses in offspring according to maternal GDM status (log-rank p<0.001).

Overall, 767 offspring from the total cohort were diagnosed with ASD during childhood. ASD diagnoses were typically established during early childhood, consistent with accepted diagnostic practices, with follow-up extending through late childhood. Univariate analysis demonstrated a significant difference between GDM severity and ASD incidence, with rates of 1.5% among offspring from GDM A2 pregnancies, 1.0% from GDM A1 pregnancies, and 0.6% from non-GDM pregnancies (p<0.001; **Table 1**). Kaplan-Meier survival analysis similarly showed difference in cumulative ASD incidence across groups (log-rank p<0.001; **Figure 2**). In Cox regression adjusted for DAG-informed baseline confounders, no statistically significant association with ASD risk was observed for either GDM A1 (aHR 1.18, 95% CI 0.83–1.66) or GDM A2 (aHR 1.56, 95% CI 0.95–2.56; **Table 1**).

## 4 Discussion

In this population-based cohort of more than 115,000 deliveries, we examined the association between GDM and the risk of ASD in offspring. Although the crude incidence of ASD was higher among offspring from GDM pregnancies, particularly GDM A2, these associations were not statistically significant after adjustment for DAG-informed baseline confounders.

While evidence linking GDM to neurodevelopmental outcomes is less consistent than that for pregestational diabetes, a subgroup of GDM-specific studies within a recent meta-analysis suggested a pooled 25% increase in ASD risk among exposed children (14). Additional meta-analysis reported a similar association, but the authors questioned the reliability of these estimates due to substantial heterogeneity across studies, inconsistent GDM diagnostic criteria, and elevated baseline ASD rates within the included populations (24). Our findings contrast with these results and are partially consistent with others who have observed that any apparent increase in risk may be confined to specific ethnic groups or to cases of GDM diagnosed earlier in pregnancy (14, 25). A sibling analysis, which helps minimize unmeasured familial confounding, likewise did not identify GDM as an independent risk factor for ASD, although its conclusions were limited by the relatively small sample size (26).

Maternal hyperglycemia is considered a plausible mechanism for altered fetal neurodevelopment, potentially acting through oxidative stress, inflammation, and epigenetic changes (10–12). However, when assessing a specific association between GDM and ASD, two important considerations should be taken into account. First, observational studies are inherently vulnerable to residual confounding, a particular concern in research on ASD given its genetic basis and the wide range of biological, environmental, and socioeconomic factors implicated in its etiology (3, 27). Second, the degree of *in-utero* exposure to hyperglycemia varies considerably and depends on both timing (gestational onset of hyperglycemia) and severity (glycemic burden). While ACOG does not define a specific lower gestational-age threshold for diagnosing GDM (28), standard screening is performed at 24–28 weeks’ gestation, including in our population, which is well beyond the critical period of early embryonic development (29). Hyperglycemia detected much earlier in pregnancy could reflect previously unrecognized type 2 diabetes rather than true gestational-onset disease. For example, Perea et al. reported an increased ASD risk among individuals diagnosed with GDM at ≤26 weeks; however, some of these cases were identified as early as 9 weeks’ gestation (30). Thus, studies describing elevated ASD risk among “early GDM” cases may partially capture the effects of pregestational diabetes rather than gestational-onset hyperglycemia. The glycemic burden of GDM, approximated by the need for pharmacologic treatment and previously suggested as a risk factor for ASD (16), did not remain significant after adjustment in our study. This attenuation may reflect the limitations of using treatment as a marker of disease severity, the effect of therapy in reducing fetal exposure, or residual differences in maternal characteristics.

Several limitations should be acknowledged. First, the observational design precludes causal inference, and residual confounding cannot be excluded. Children born to parents with GDM may have increased healthcare contact, potentially increasing the likelihood of ASD detection independent of true neurodevelopmental risk. Second, detailed measures of glycemic control and treatment modality were unavailable. Residual confounding related to obesity cannot be excluded, as obesity was modeled as a binary variable rather than continuous BMI. Finally, differences in ethnic composition between study groups may also represent a potential limitation, as ethnicity is associated with metabolic risk and ASD prevalence, and residual confounding may persist despite adjustment. Another limitation for the study is the limited number of ASD events among the GDM A2 group which possibly reduced statistical power and resulted in wide confidence intervals. The study’s strengths include its large sample size and the integration of follow-up data from both hospital and outpatient settings, which enhances case identification. The study population is served by accessible, centralized healthcare, increasing the chances of timely and consistent diagnoses. Furthermore, by focusing solely on GDM and excluding pregestational diabetes, the study allowed a clearer assessment of the implications of pregnancy-related hyperglycemia.

In conclusion, our findings indicate that GDM and its subtypes, despite their known associations with obstetric and neonatal complications, do not appear to increase the long-term risk of ASD in offspring after full adjustment. However, it remains possible that early-onset or poorly controlled GDM may exert subtle effects on fetal neurodevelopment that are not detectable in our regional cohort, despite comprehensive and centralized healthcare coverage. As understanding of the early determinants of neurodevelopment continues to evolve, appropriate management of GDM and other health conditions during pregnancy remains important, not only to reduce short-term risks, but also with attention to broader lifelong health implications.

## Data Availability

The original contributions presented in the study are included in the article/**Supplementary Material**. Further inquiries can be directed to the corresponding author.

## Supplementary material

Supplementary Figure 1 Directed acyclic graph (DAG) illustrating the assumed causal structure between gestational diabetes mellitus (GDM) and autism spectrum disorder (ASD) in offspring. Baseline confounders include maternal age, ethnicity, obesity, smoking, and fertility treatment. Child sex and birth year were included as predictors of ASD diagnosis. Surveillance represents a potential unmeasured detection pathway. The DAG was used to define the adjustment set for the primary Cox regression model.

Supplementary Figure 2 Flow diagram of study cohort selection. The diagram illustrates the selection of the birth cohort included in the analysis. All deliveries at Soroka University Medical Center between 2005 and 2017 were considered eligible. Pregnancies complicated by pregestational diabetes, diabetes in pregnancy, major congenital anomalies, or multiple gestations were excluded. The final study cohort consisted of 115,063 deliveries and was categorized into pregnancies without gestational diabetes mellitus (GDM), diet-controlled GDM (GDM A1), and pharmacologically treated GDM (GDM A2). Follow-up for autism spectrum disorder (ASD) diagnoses was conducted through 2021 using centralized community and hospital records.
