# Supplementary figures and images for: Gestational diabetes mellitus and the risk of autism spectrum disorder in offspring: a population-based retrospective cohort study
Source: Front Clin Diabetes Healthc. 2026 Feb 23;7:1754571. doi: 10.3389/fcdhc.2026.1754571 (PMC12968014; doi:10.3389/fcdhc.2026.1754571)

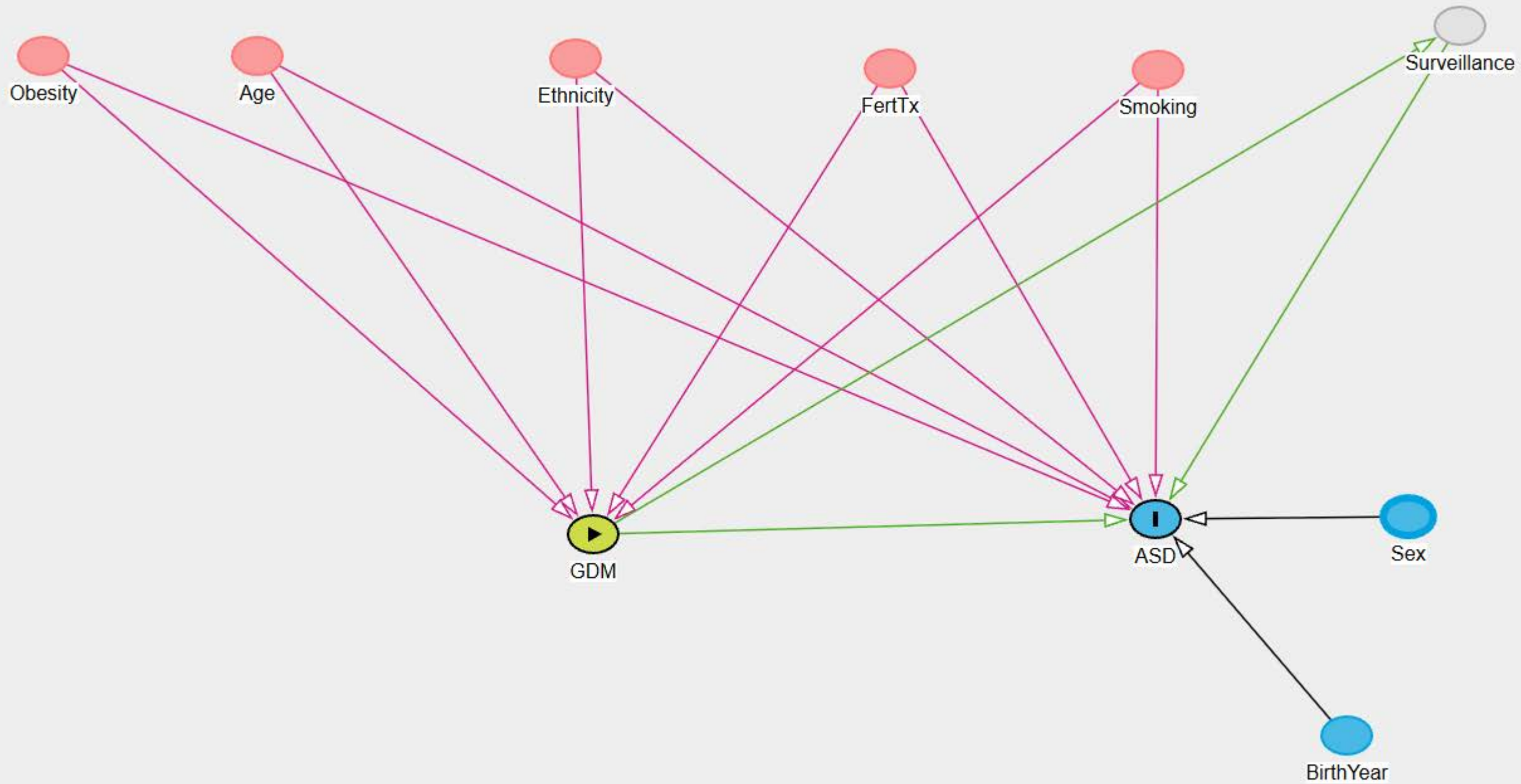

Supplement: Supplementary Figure 1 — Directed acyclic graph (DAG) illustrating the assumed causal structure between gestational diabetes mellitus (GDM) and autism spectrum disorder (ASD) in offspring. Baseline confounders include maternal age, ethnicity, obesity, smoking, and fertility treatment. Child sex and birth year were included as predictors of ASD diagnosis. Surveillance represents a potential unmeasured detection pathway. The DAG was used to define the adjustment set for the primary Cox regression model. [file Image1.pdf]
