# Supplementary material for: Gestational diabetes mellitus and the risk of autism spectrum disorder in offspring: a population-based retrospective cohort study
Source: Front Clin Diabetes Healthc. 2026 Feb 23;7:1754571. doi: 10.3389/fcdhc.2026.1754571 (PMC12968014; doi:10.3389/fcdhc.2026.1754571)
Supplement: Supplementary Figure 2 — Flow diagram of study cohort selection. The diagram illustrates the selection of the birth cohort included in the analysis. All deliveries at Soroka University Medical Center between 2005 and 2017 were considered eligible. Pregnancies complicated by pregestational diabetes, diabetes in pregnancy, major congenital anomalies, or multiple gestations were excluded. The final study cohort consisted of 115,063 deliveries and was categorized into pregnancies without gestational diabetes mellitus (GDM), diet-controlled GDM (GDM A1), and pharmacologically treated GDM (GDM A2). Follow-up for autism spectrum disorder (ASD) diagnoses was conducted through 2021 using centralized community and hospital records. [file Image2.pdf]

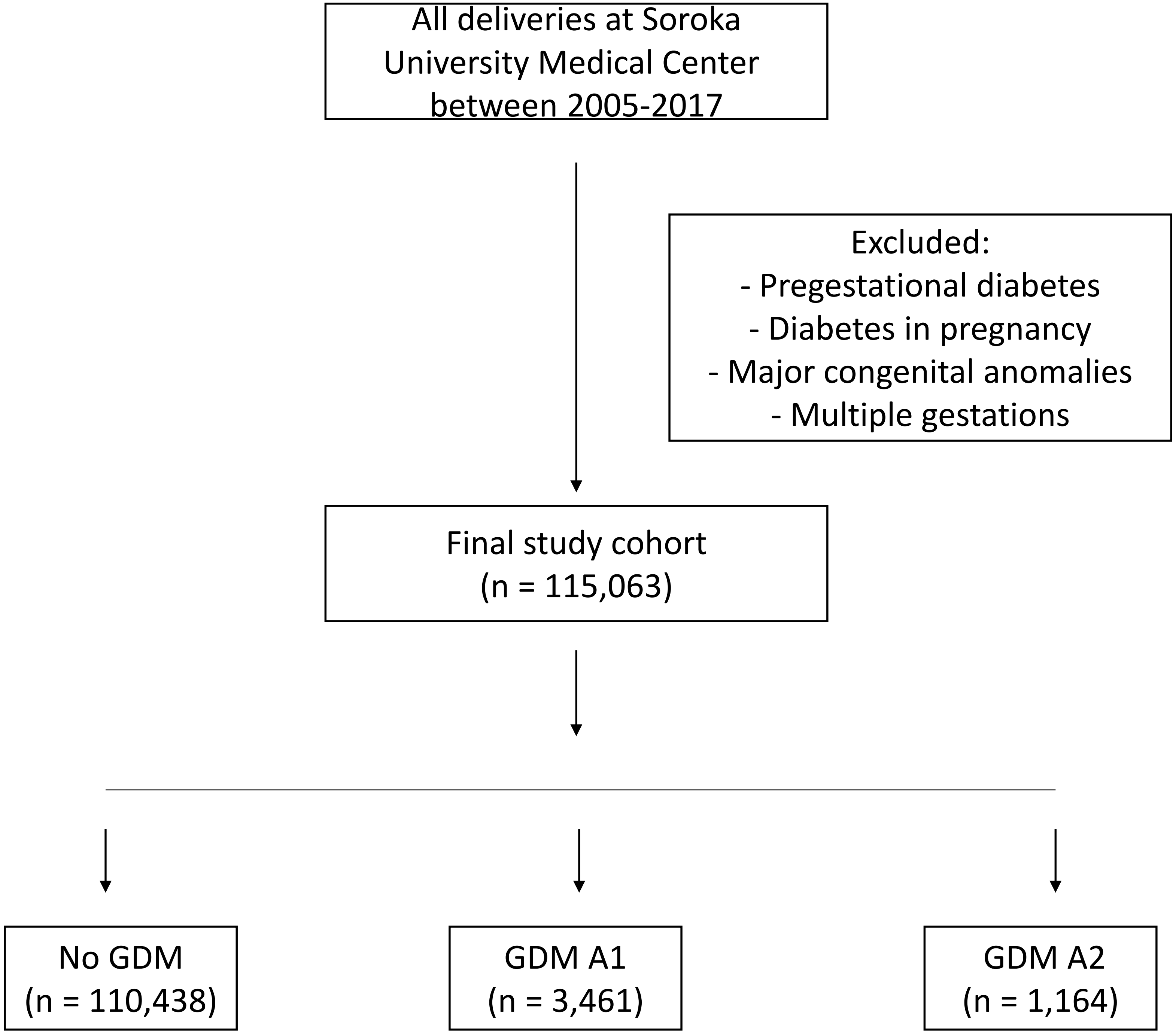
All deliveries at Soroka University Medical Center between 2005-2017
Excluded:
- Pregestational diabetes
- Diabetes in pregnancy
- Major congenital anomalies
- Multiple gestations
Final study cohort (n = 115,063)
No GDM (n = 110,438)
GDM A1 (n = 3,461)
GDM A2 (n = 1,164)
